# Deficiency of SCAMP5 Triggers Pancreatic β‐Cell Secretory Dysfunction and Apoptosis

**DOI:** 10.1002/advs.202503072

**Published:** 2025-09-15

**Authors:** Yingqi Zhang, Donglan Tang, Chenxi Yang, Chao Lin, Yingyue Yuan, Meiying He, Yiying Chen, Zhuo Mao, Ying Ying, Xiaosong Ma, Xiangchen Kong

**Affiliations:** ^1^ Shenzhen University Diabetes Institute Shenzhen Key Laboratory of Metabolism and Cardiovascular Homeostasis Shenzhen University Medical School Shenzhen 518060 China; ^2^ Department of Orthopaedic and Reconstructive Surgery/Pediatric Orthopaedics South China Hospital Medical School Shenzhen University Shenzhen 518116 China

**Keywords:** β‐cell apoptosis, ChREBP, insulin secretion, SCAMP5, VDAC1

## Abstract

The late stage of type 2 diabetes is characterized by secretory dysfunction and increased β‐cell apoptosis, but the underlying mechanisms are not fully understood. The expression and function of secretory carrier membrane protein 5 (SCAMP5) in β‐cells are unclear. The aim is to explore the role of SCAMP5 in diabetic β‐cell failure. SCAMP5 expression is reduced in β‐cells under diabetic conditions. Notably, SCAMP5 deficiency diminishes insulin secretion, which is involved in reduced Ca_V_1.2 expression. Additionally, decreased SCAMP5 triggers β‐cell apoptosis, suggesting the anti‐apoptotic role of SCAMP5 in β‐cells. Mechanistically, SCAMP5 downregulates the protein expression of voltage‐dependent anion channel (VDAC1) and interacts with it, thereby repressing VDAC1‐recruited Bax to mitochondria, thus inhibiting the release of cytochrome c from mitochondria to the cytoplasm, culminating in preventing β‐cell apoptosis. Furthermore, hyperglycemia‐activated carbohydrate‐responsive element‐binding protein (ChREBP) epigenetically represses SCAMP5 expression by reducing trimethylation of histone H3 at lysine 4 (H3K4me3) within the *Scamp5* promoter. These findings highlight the essential role of the ChREBP‐controlled SCAMP5 in β‐cell insulin secretion and apoptosis, revealing a previously unrecognized mechanism underlying the β‐cell failure in diabetes.

## Graphical abstract

This study demonstrates that hyperglycemia in diabetes activates ChREBP, which epigenetically represses SCAMP5 expression in pancreatic β‐cells. This SCAMP5 deficiency impairs insulin secretion by downregulating the calcium channel Ca_V_1.2. Furthermore, reduced SCAMP5 triggers β‐cell apoptosis by stabilizing VDAC1 and enhancing its interaction with Bax, leading to mitochondrial cytochrome c release. Thus, SCAMP5 is crucial for β‐cell function and survival.

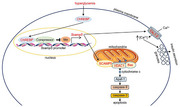

## 1 Introduction

The late stage of type 2 diabetes (T2D) is characterized by hyperglycemia, which results from the inability of pancreatic β‐cells to secrete sufficient insulin owing to β‐cell failure driven by secretory dysfunction and apoptosis.^[^ ^1^, ^2^ ^]^ Although substantial efforts have focused on understanding the mechanisms of β‐cell failure in T2D,^[^ ^3^ ^]^ the detailed mechanisms remain elusive. Therefore, the identification of the key regulators of β‐cell failure to uncover the mechanisms underlying β‐cell dysfunction would offer a new therapeutic target for T2D.

ChREBP is a glucose‐sensitive transcription factor that plays a crucial role in glucose metabolism.^[^ ^4^ ^]^ It consists of different functional domains, including the N‐terminal glucose‐sensing module with the low glucose inhibitory domain (LID) and the glucose‐activated conserved element (GRACE), as well as the C‐terminal regions.^[^ ^4^ ^]^ ChREBP regulates the expression of target genes upon high glucose activation, a process via binding to carbohydrate response elements (ChoRE) on the promoter of the target gene and then causing a change in the histone modification at the promoter region, thereby regulating the transcriptional expression of the target gene.^[^ ^5^ ^]^ Notably, it is activated in diabetic β‐cells and mediates glucotoxicity‐induced apoptosis of β‐cells.^[^ ^6^ ^]^ However, the downstream target genes regulated by ChREBP remain largely unidentified.

DNA survey reveals that there is a ChoRE in the promoter of SCAMP5. It is a member of the SCAMP family, which consists of five different proteins (SCAMP1‐5).^[^ ^7^ ^]^ These proteins are integral membrane proteins that are involved in the trafficking of membrane‐bound cargo within cells. SCAMP5 is implicated in the regulation of neuronal vesicular transportation, vesicle exocytosis, and endocytosis.^[^ ^7^ ^]^ It plays a crucial role in a variety of neurological conditions, such as autism^[^ ^8^ ^]^ and juvenile epilepsy.^[^ ^9^ ^]^ However, the role of SCAMP5 in pancreatic β‐cells remains unclear. Here, we show that SCAMP5 expression is reduced within β‐cells in diabetogenic situations. Decreased SCAMP5 facilitates β‐cell secretory dysfunction and apoptosis. Furthermore, SCAMP5 expression is epigenetically controlled by ChREBP. Hyperglycemia is likely to activate ChREBP, thereby inhibiting SCAMP5 expression and subsequently causing β‐cell dysfunction.

## 2 Results

### 2.1 The Expression of SCAMP5 Is Decreased in Diabetic β‐Cells

**Figure 1:**
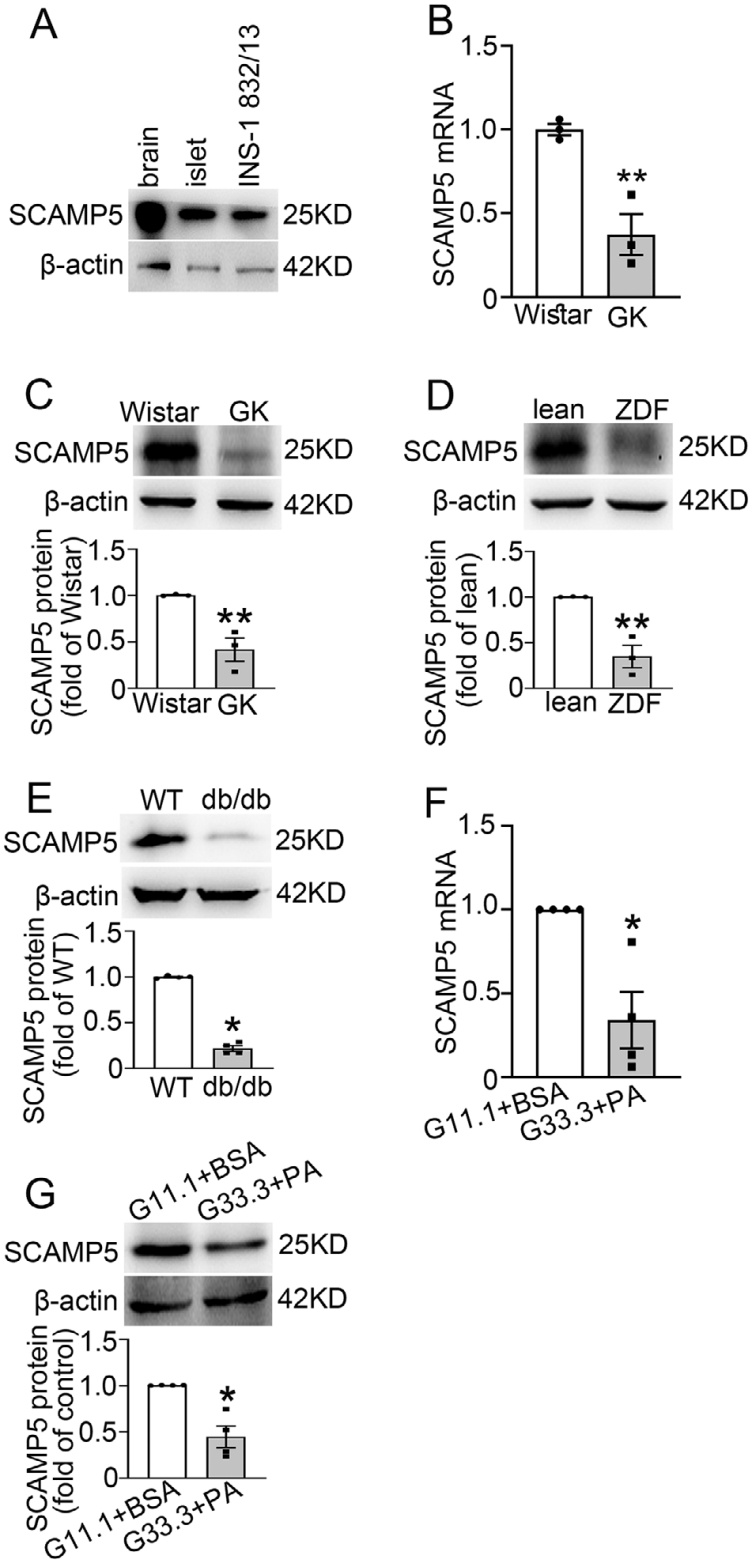
SCAMP5 expression is reduced in β‐cells under diabetogenic situations. A) The protein expression of SCAMP5 in mouse brain, mouse islets, and INS‐1 832/13 cells. β‐actin was used as a loading control. The B) mRNA and C) protein expression of SCAMP5 in islets isolated from Wistar and GK rats. Data are means ± S.E.M., *n* = 3 rats per group. **P< 0.01. D) The protein levels of SCAMP5 in islets from lean and ZDF rats. Data are means ± S.E.M. of three rats per group. **P< 0.01. E) The protein levels of SCAMP5 in islets isolated from 20‐week‐old wild‐type (WT) and db/db mice. Data are means ± S.E.M. of four mice per group. *P< 0.05. The F) mRNA and G) protein expression of SCAMP5 in INS‐1 832/13 cells treated with 11.1 mm glucose and 10% BSA (G11.1+BSA) or 33.3 mm glucose and 0.4 mm palmitic acid (G33.3+PA) for 14 h. Data are means ± S.E.M., *n* = 4. *P< 0.05. Statistical analyses were performed with an unpaired two‐tailed t‐test (B–D) or Mann‐Whitney *U* test (E–G).

We first confirmed that SCAMP5 is expressed in pancreatic β‐cells, as demonstrated by comparable protein levels in β‐cells and neurons (**Figure** **1**A). Subsequently, we examined SCAMP5 expression in different diabetogenic situations. Non‐obese diabetic Goto‐Kakizaki (GK) rat islets exhibited significantly reduced mRNA and protein levels of SCAMP5, respectively, compared with those in Wistar rats (Figure 1B,C). Similar observations were also made in obese Zucker Diabetic Fatty (ZDF) rats (Figure 1D), db/db mice (Figure 1E), and INS‐1 832/13 cells exposed to glucolipotoxicity conditions (Figure 1F,G). These data suggest that SCAMP5 expression is reduced in rodent β‐cells under diabetic conditions. However, SCAMP5 expression in diabetic patient islets remains to be investigated in future studies.

### 2.2 SCAMP5 Is Required for Glucose‐Stimulated Insulin Secretion in β‐Cells

**Figure 2:**
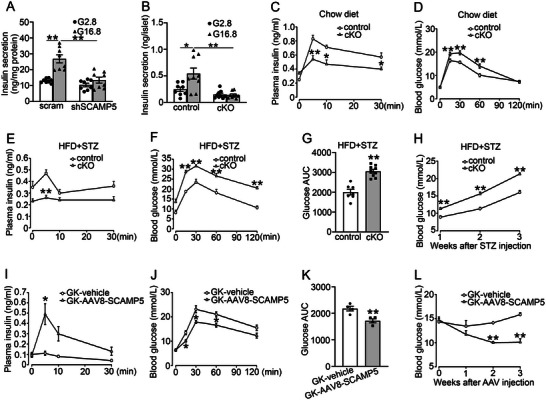
SCAMP5 regulates insulin secretion in β‐cells. A) The scramble and shSCAMP5 INS‐1 832/13 cells were stimulated with 2.8 mmol L^−1^ (G2.8) or 16.8 mmol L^−1^ (G16.8) glucose for 30 min. The insulin levels were determined by the ELISA kit. Data are means ± S.E.M., *n* = 8. **P<0.01. B) The islets isolated from control and *Scamp5* cKO mice were stimulated with G2.8 or G16.8 for 30 min. Data are means ± S.E.M., *n* = 8–9. *P < 0.05, **P < 0.01. C) Plasma insulin in control and cKO mice. Data are means ± S.E.M. of four mice per group. *P < 0.05, **P < 0.01. D) IPGTT was performed in control and cKO mice. After overnight fasting, the mice were intraperitoneally injected with 2.0 g kg^−1^ glucose. *n* = 11–20 mice per group. **P< 0.01. E–H) After one month of feeding with HFD, the control and *Scamp5* cKO mice were intraperitoneally injected with 50 mg kg^−1^ body weight STZ for five consecutive days. E) Plasma insulin levels were measured in control and cKO mice after intraperitoneal glucose injection (2 g kg^−1^ body weight). Data are means ± S.E.M., *n* = 5–6 mice per group. **P< 0.01. F) Plasma glucose levels were determined during IPGTT in control and cKO mice. Data are means ± S.E.M., *n* = 7–9 mice in each group. **P < 0.01. G) The area under the curves (AUC) was calculated for glucose in (F). Data are means ± S.E.M., *n* = 7–9, **P < 0.01. H) Blood glucose in control and cKO mice after STZ injection. Data are means ± S.E.M., *n* = 3–5, **P< 0.01. I) Plasma insulin in GK‐vehicle and GK‐AAV8‐SCAMP5 rats. Data are means ± S.E.M., *n* = 3‐4 rats per group. *P< 0.05. J) Plasma glucose levels were measured during IPGTT in GK‐vehicle and GK‐AAV8‐SCAMP5 rats. Data are means ± S.E.M. of four rats per group. *P<0.05. K) The area under the curves (AUC) was calculated for glucose in (J). Data are means ± S.E.M., *n* = 4, **P< 0.01. L) Plasma glucose in GK‐vehicle and GK‐AAV8‐SCAMP5 rats. Data are means ± S.E.M. of three rats in each group. **P< 0.01. Statistical analyses were performed using A, B, D, F, I) the Mann‐Whitney *U* test or C,E,G,H, J–L) unpaired t‐test.

To explore the impact of reduced SCAMP5 on insulin secretion, we generated the scramble and SCAMP5 knockdown (shSCAMP5) INS‐1 832/13 cells (Figure S1, Supporting Information) and assessed insulin secretion in these cells. Notably, shSCAMP5 impaired glucose‐stimulated insulin secretion (GSIS) (**Figure** **2**A). To confirm this finding in vivo, we generated β‐cell‐specific *Scamp5* knockout mice (*Scamp5^flox/flox^‐Ins2‐Cre*, cKO) alongside control mice (*Scamp5^flox/flox^*) and confirmed successful deletion of *Scamp5* (Figure S2A, Supporting Information). Consistently, *Scamp5* cKO diminished GSIS both in vitro (Figure 2B) and in vivo (Figure 2C), coinciding with impaired glucose control (Figure 2D). Additionally, to rule out potentially confounding effects of the *Ins2‐Cre* transgene, SCAMP5‐deficiency‐induced glucose intolerance was further evaluated in *Scamp5^flox/flox^‐Ins2‐Cre* and *Scamp5^flox/+^‐Ins2‐Cre* mice (Figure S2B, Supporting Information), and corroborated by findings obtained from tamoxifen‐induced β‐cell‐specific *Scamp5* knockout mice (*Scamp5^flox/flox^‐Pdx1‐Cre*) and control mice (Figure S2C, Supporting Information). Nonetheless, cKO and control mice displayed comparable non‐fasting blood glucose levels (Figure S2D, Supporting Information) and body weight (Figure S2E, Supporting Information). To investigate the potential role of SCAMP5 in underlying β‐cell dysfunction associated with T2D, we subjected control and cKO mice to a high‐fat diet (HFD) in combination with low‐dose streptozotocin (STZ), which mimics late‐stage human T2D. In this experimental setting, cKO mice displayed lower plasma insulin levels (Figure 2E), impaired glucose tolerance (Figure 2F,G), and higher blood glucose levels (Figure 2H).

To confirm the essential role of SCAMP5 in insulin secretion, we generated adeno‐associated virus serotype 8 encoding SCAMP5 (AAV8‐SCAMP5) under the β‐cell‐specific insulin1 promoter to specifically target pancreatic islets. The AAV8‐SCAMP5 or control virus (vehicle) was delivered into diabetic GK rats via bile duct injection. This administration of AAV8‐SCAMP5 enhanced SCAMP5 protein expression in the islets (Figure S3A, Supporting Information), but not in other tissues (Figure S3B, Supporting Information). Importantly, GK‐AAV8‐SCAMP5 rats demonstrated higher plasma insulin levels (Figure 2I), improved glucose tolerance (Figure 2J,K), and progressively decreased blood glucose levels (Figure 2L) compared to GK‐vehicle rats, suggesting the remission of diabetes.

### 2.3 SCAMP5 Regulates Ca_V_1.2 Expression in β‐Cells

**Figure 3:**
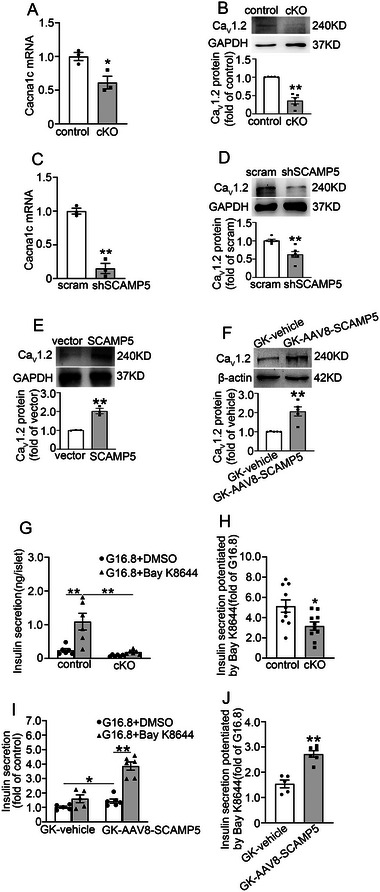
SCAMP5 regulates Ca_V_1.2 expression in β‐cells. A) The mRNA and B) protein expression of Ca_V_1.2 (*Cacna1c*) in islets isolated from control and *Scamp5* cKO mice. Data are means ± S.E.M., *n* = 3–5 mice per group. *P< 0.05, **P< 0.01. C) The mRNA and D) protein expression of Ca_V_1.2 in scramble and shSCAMP5 INS‐1 832/13 cells. Data are means ± S.E.M., *n* = 3–6. **P< 0.01. E) The protein levels of Ca_V_1.2 in vector and SCAMP5‐overexpressing INS‐1 832/13 cells. Data are means ± S.E.M., *n* = 3. **P < 0.01. F) The protein levels of Ca_V_1.2 in islets from GK‐vehicle and GK‐AAV8‐SCAMP5 rats. Data are means ± S.E.M., *n* = 5. **P< 0.01. G,H) The islets isolated from control and *Scamp5* cKO mice were stimulated with G16.8 in the presence or absence of 1 µmol L^−1^ Bay K8644 for 30 min. Insulin levels were examined with G) an ELISA kit. H)Statistical analysis of Bay K8644‐induced fold increase of insulin secretion in (G). Data are means ± S.E.M., *n* = 6–10. *P < 0.05, **P<0.01. I,J) Similar to G,H), the islets were isolated from GK‐vehicle and GK‐AAV8‐SCAMP5 rats. Data are means ± S.E.M., *n* = 5–6. *P< 0.05, **P<0.01. Data analysis was conducted using A,C,D,E,H,J) an unpaired t‐test or B,F,G,I) Mann‐Whitney *U* test.

To explore the mechanisms through which SCAMP5 influences insulin secretion, we assessed its effects on the expression of Ca_V_1.2, the pore‐forming subunit of L‐type voltage‐dependent calcium channels, which play an essential role in insulin secretion.^[^ ^10^ ^]^ Intriguingly, *Scamp5* cKO islets exhibited a significant reduction in Ca_V_1.2 mRNA (**Figure** **3**A) and protein (Figure 3B) expression. Consistent results were obtained in shSCAMP5 INS‐1 832/13 cells (Figure 3C,D). In contrast, SCAMP5 overexpression (Figure S4A, Supporting Information) upregulated Ca_V_1.2 expression in INS‐1 832/13 cells (Figure 3E), an observation that was further validated in GK‐AAV8‐SCAMP5 rat islets (Figure 3F). To assess the functional impact of SCAMP5 on Ca_V_1.2 activity, we analyzed insulin secretion potentiated by Bay K8644, a positive allosteric modulator of L‐type calcium channels. This revealed that Bay K8644‐potentiated insulin secretion was diminished in *Scamp5* cKO islets (Figure 3G,H), but enhanced in GK‐AAV8‐SCAMP5 islets (Figure 3I,J). These findings highlight an important role of Ca_V_1.2 in SCAMP5‐mediated regulation of insulin secretion.

### 2.4 A Deficiency of SCAMP5 Promotes β‐Cell Apoptosis

**Figure 4:**
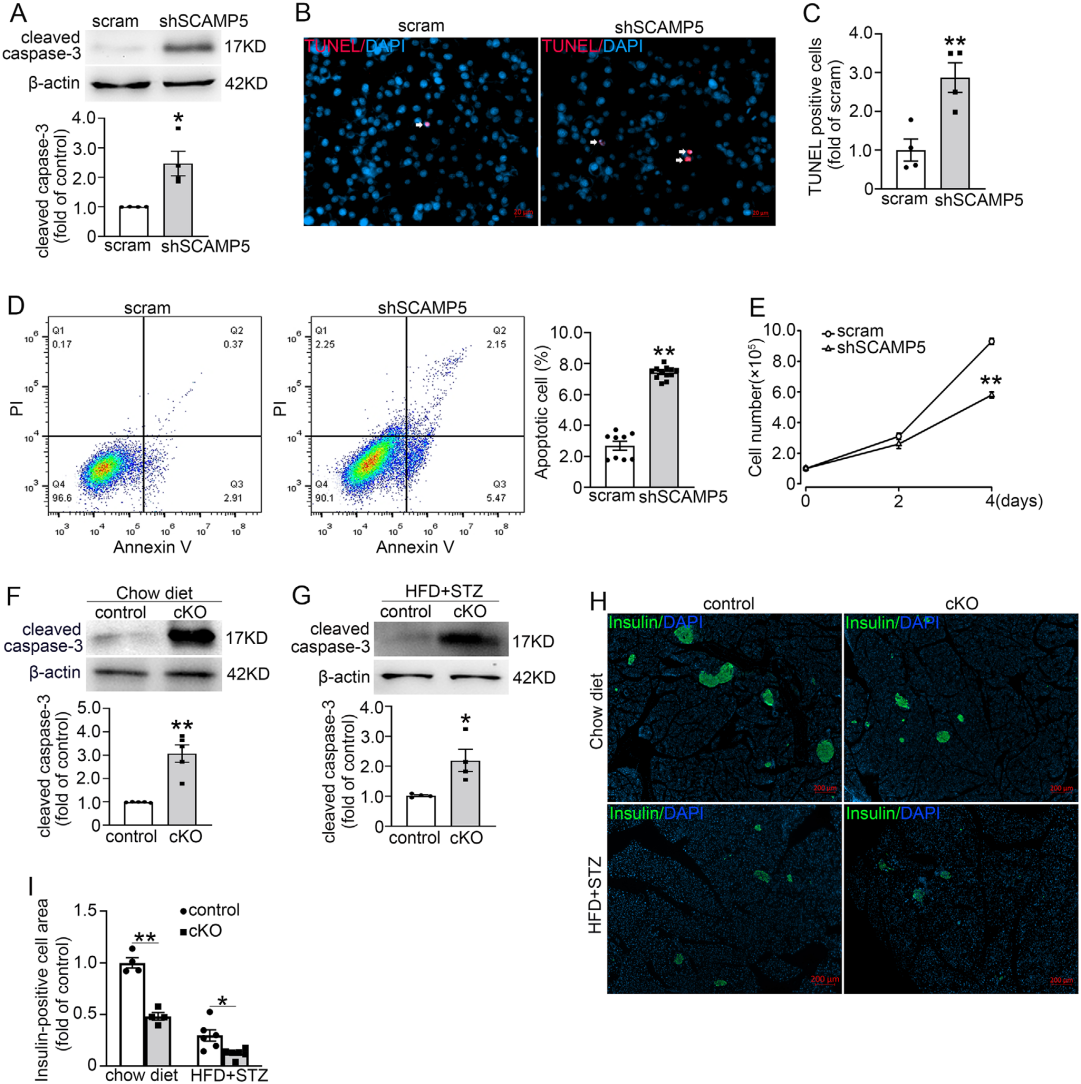
SCAMP5 deficiency facilitates β‐cell apoptosis. A) Western blot was conducted to determine the levels of cleaved caspase‐3 in scramble and shSCAMP5 INS‐1 832/13 cells. β‐actin was used as a loading control. Data are means ± S.E.M., *n* = 4. *P < 0.05. B) Cell apoptosis was identified by TUNEL staining in scramble and shSCAMP5 INS‐1 832/13 cells. Arrowheads indicate apoptotic cells. Scale bar, 20 µm. C) Quantification of the TUNEL‐positive cell rate in (B). Data are means ± S.E.M. from four independent experiments. **P< 0.01. D) Cell apoptosis was examined via flow cytometry in scramble and shSCAMP5 INS‐1 832/13 cells incubated with PI and Annexin V. Data are means ± S.E.M., *n* = 9–12. **P< 0.01. E) The cell number was determined in scramble and shSCAMP5 INS‐1 832/13 cells at the indicated time. Values are means ± S.E.M. of three independent experiments. **P< 0.01. F) Western blot was performed to evaluate the levels of cleaved caspase‐3 in islets from control and *Scamp5* cKO mice. β‐actin was utilized as a loading control. Data are means ± S.E.M. of five mice per group. **P< 0.01. G) The protein levels of cleaved caspase‐3 were analyzed in islets from control and cKO mice treated with HFD and STZ. β‐actin was used as a loading control. Data are means ± S.E.M. of four mice per group. *P < 0.05. H) Immunofluorescence of insulin (green) and DAPI (blue) in pancreata of control and cKO mice treated with chow diet or HFD and STZ. Scale bar, 200 µm. I) Quantification of insulin‐positive cell area in (H). Data are means ± S.E.M., *n* = 4–6 mice per group. *P < 0.05, **P < 0.01. Statistical analyses were performed using A,D,F) the Mann‐Whitney *U* test or C,E,G,I) unpaired t‐test.

To investigate the impact of SCAMP5 downregulation on β‐cell apoptosis, we assessed apoptosis in scramble and shSCAMP5 INS‐1 832/13 cells. As shown in **Figure** **4**A, shSCAMP5 resulted in an increase in cleaved caspase‐3 levels compared to the scramble cells. The pro‐apoptotic effect of shSCAMP5 was confirmed by TUNEL assay (Figure 4B,C) and flow cytometry (Figure 4D). Consequently, shSCAMP5 reduced the INS‐1 832/13 cell number (Figure 4E).

The cell apoptosis was further evaluated in the control and *Scamp5* cKO mice under both chow diet and diabetogenic conditions. Remarkably, cleaved caspase‐3 levels were significantly elevated in cKO islets across both conditions (Figure 4F,G), suggesting that SCAMP5 deficiency promotes β‐cell apoptosis. In line with the increased apoptosis in *Scamp5* cKO islets, the insulin‐positive cell area was reduced in KO mice compared with that of control mice (Figure 4H,I).

### 2.5 Increased SCAMP5 Inhibits β‐Cell Apoptosis

**Figure 5:**
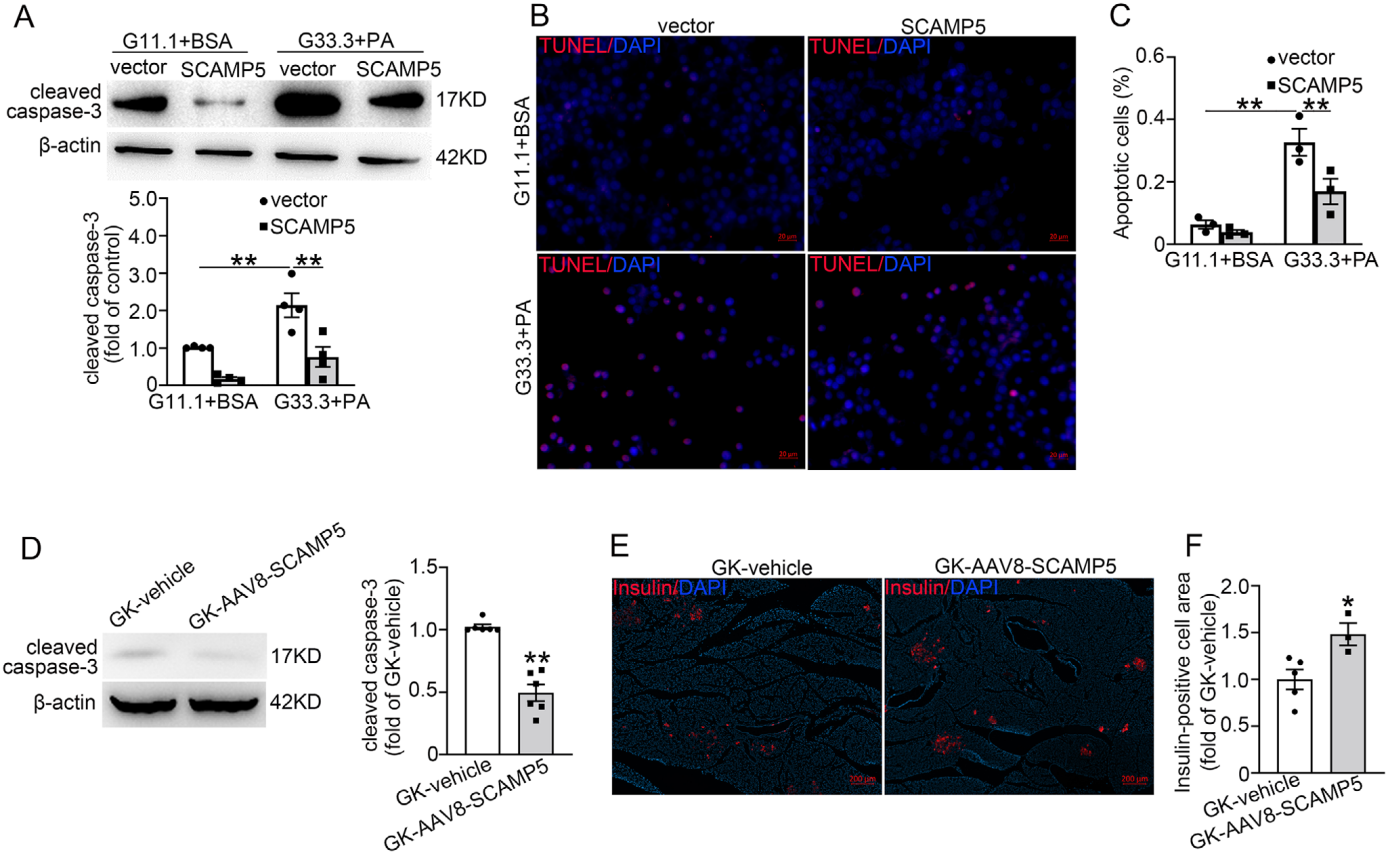
Overexpression of SCAMP5 suppresses β‐cell apoptosis. A–C) The vector and SCAMP5‐overexpressing INS‐1 832/13 cells were cultivated in a medium containing 11.1 mm glucose and 10% BSA (G11.1+BSA) or 33.3 mm glucose and 0.4 mm palmitic acid (G33.3+PA) for 14 h. Cleaved caspase‐3 levels were assessed by A) Western blot. The apoptotic cells were monitored through B) TUNEL staining assay, and C) the ratio of apoptotic cells was analyzed. Data are means ± S.E.M., *n* = 4 in (A) or 3 in (C). **P< 0.01. D) The protein levels of cleaved caspase‐3 in islets from GK‐vehicle and GK‐AAV8‐SCAMP5 rats. β‐actin was used as a loading control. Data are means ± S.E.M., *n* = 6 rats per group. **P < 0.01. E) Immunofluorescence of insulin (red) in pancreata of GK rats subjected to intraductal injection of vehicle or AAV8‐SCAMP5 virus. Nuclei were stained with DAPI. Scale bar, 200 µm. F) Quantification of the insulin‐positive cell area in (E). Data are means ± S.E.M., *n* = 3–5 rats per group. *P < 0.05. Statistical tests included A,C) one‐way ANOVA with LSD post‐hoc test, D) Mann‐Whitney *U* test, or F) unpaired t‐test.

To confirm the anti‐apoptotic effect of SCAMP5, we further investigated the effect of SCAMP5 upregulation on β‐cell apoptosis both in vitro and in vivo. The vector and SCAMP5‐overexpressing INS‐1 832/13 cells were exposed to 33.3 mm glucose medium with 0.4 mm palmitic acid (G33.3+PA) or 11.1 mm glucose medium with 10% BSA (G11.1+BSA) for 14 h before analyzing apoptosis. The results showed that G33.3 and PA exposure resulted in significantly increased cell apoptosis, as demonstrated by increased cleaved caspase‐3 levels and TUNEL‐positive cells; however, SCAMP5 overexpression mitigated these effects (**Figure** **5**A–C). Similarly, SCAMP5 overexpression diminished STZ‐induced cell apoptosis (Figure S4B, Supporting Information). The protective effect of increased SCAMP5 was also investigated in GK‐AAV8‐SCAMP5 rats. AAV8‐SCAMP5 administration reduced cleaved caspase‐3 levels in GK rat islets (Figure 5D). Accordingly, the insulin‐positive cell area was greater in GK‐AAV8‐SCAMP5 rats than in control rats (Figure 5E,F).

### 2.6 SCAMP5 Deficiency Triggers Cytochrome C Release from Mitochondria to the Cytoplasm by Regulating VDAC1

**Figure 6:**
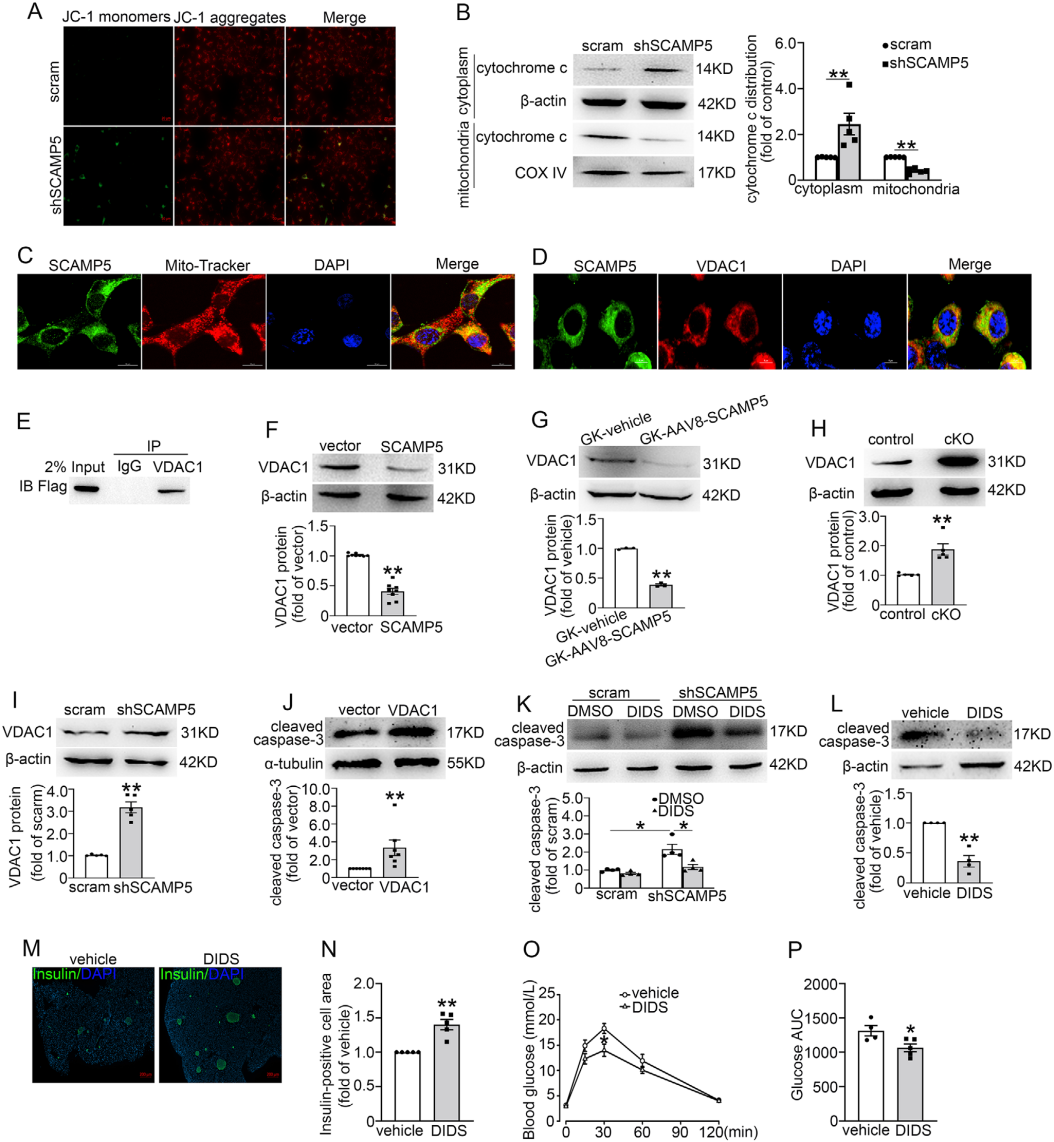
SCAMP5 regulates cytochrome c release from mitochondria via modulation of VDAC1. A) The △*Ψm* of scramble and shSCAMP5 INS‐1 832/13 cells was determined by JC‐1 staining. Green fluorescence indicated JC‐1 monomers. Scale bar, 20 µm. B) The subcellular distribution of cytochrome c in scramble and shSCAMP5 INS‐1 832/13 cells was determined by Western blot. β‐actin and COX IV were used as internal controls of cytosolic and mitochondrial protein, respectively. Data are means ± S.E.M. from five independent experiments. **P<0.01. C) Distribution of SCAMP5 (green) on mitochondria (red) in INS‐1 832/13 cells. Scale bar, 10 µm. D) Co‐localization of SCAMP5 (green) and VDAC1 (red) in INS‐1 832/13 cells. Scale bar, 5 µm. E) The interaction between SCAMP5 and VDAC1 in INS‐1 832/13 cells overexpressing Flag‐tagged SCAMP5 was analyzed by immunoprecipitation using an anti‐VDAC1 antibody, followed by Western blot analysis with an anti‐Flag antibody. F) VDAC1 protein levels in vector and SCAMP5‐overexpressing INS‐1 832/13 cells were determined by Western blot. β‐actin was used as a loading control. Data are means ± S.E.M. of eight independent experiments. **P < 0.01. G) The protein expression of VDAC1 in islets from GK‐vehicle and GK‐AAV8‐SCAMP5 rats. Data are means ± S.E.M. of three rats per group. **P< 0.01. H) VDAC1 protein expression in islets from the control and *Scamp5* cKO mice. Data are means ± S.E.M. of five mice per group. **P< 0.01. I) VDAC1 protein level in scramble and shSCAMP5 INS‐1 832/13 cells. Data are means ± S.E.M. of five independent experiments. **P< 0.01. J) The protein levels of cleaved caspase‐3 in vector and VDAC1‐overexpressing INS‐1 832/13 cells. α‐tubulin was used as a loading control. Data are means ± S.E.M., *n* = 7. **P < 0.01. K) The protein levels of cleaved caspase‐3 in scramble and shSCAMP5 INS‐1 832/13 cells treated with 200 µm DIDS for 2 h. β‐actin was used as a loading control. Data are means ± S.E.M., *n* = 4. *P <0.05. L–P) *Scamp5* cKO mice were intraperitoneally injected with saline or 50 mg kg^−1^ body weight DIDS for two weeks. L) The protein levels of cleaved caspase‐3 in islets isolated from *Scamp5* cKO mice treated with saline or DIDS. Data are mean ± S.E.M., *n* = 4 mice per group. **P < 0.01. M) Immunofluorescence of insulin (green) and DAPI (blue) in pancreata of *Scamp5* cKO mice treated with saline or DIDS. Scale bar, 200 µm. N) Quantification of the insulin‐positive cell area in (M). Data are mean ± S.E.M. from five mice per group. **P < 0.01. O) Plasma glucose levels were measured during IPGTT in control and *Scamp5* cKO mice. Data are mean ± S.E.M., *n* = 4–6. *P < 0.05. P)The area under the curves (AUC) was calculated for glucose in (O). Data are mean ± S.E.M., *n* = 4–5, *P < 0.05. Statistical comparisons were carried out using B,F,I–K,N) the Mann‐Whitney *U* test G,H,L,O,P) or unpaired t‐test.

To understand how SCAMP5 regulates β‐cell apoptosis, we examined the impact of shSCAMP5 on the membrane potential of mitochondria (*ΔΨm*). The shSCAMP5 INS‐1 832/13 cells exhibited higher green fluorescence, indicating a reduction in *ΔΨm* (**Figure** **6**A). Consistently, shSCAMP5 promoted the release of cytochrome c from mitochondria to the cytoplasm (Figure 6B), thereby activating caspase‐9 (Figure S5A, Supporting Information). To elucidate the reason for SCAMP5 affecting Δ*Ψm*, we investigated its mitochondrial localization in INS‐1 832/13 cells. This revealed that SCAMP5 is indeed localized to mitochondria (Figure 6C).

Voltage‐dependent anion channel 1 (VDAC1), located in the outer mitochondrial membrane, has been shown to mediate the release of cytochrome c from mitochondria.^[^ ^11^ ^]^ We hypothesized that VDAC1 could participate in SCAMP5‐regulated cytochrome c release. We, therefore, investigated the interaction between SCAMP5 and VDAC1. Immunofluorescence results demonstrated colocalization of SCAMP5 with VDAC1 in INS‐1 832/13 cells (Figure 6D). To further corroborate this observation, we conducted a co‐immunoprecipitation (Co‐IP) assay to evaluate the interaction between SCAMP5 and VDAC1 in INS‐1 832/13 cells overexpressing Flag‐tagged SCAMP5 (Figure S4A, Supporting Information). Specifically, immunoprecipitation (IP) was carried out using a VDAC1 antibody, and the bound SCAMP5 was subsequently detected using a Flag antibody. These results confirmed that SCAMP5 interacts with VDAC1 (Figure 6E).

We further examined the impact of SCAMP5 on VDAC1 expression. Notably, overexpression of SCAMP5 reduced VDAC1 protein expression compared to vector control cells (Figure 6F). Similar observations were also made in GK‐AAV8‐SCAMP5 rat islets (Figure 6G). By contrast, SCAMP5 deficiency enhanced VDAC1 protein levels (Figure 6H,I), albeit shSCAMP5 did not affect VDAC1 mRNA levels (Figure S5B, Supporting Information). Intriguingly, the proteasome inhibitor MG‐132 diminished the increased VDAC1 protein levels induced by shSCAMP5 (Figure S5C, Supporting Information). These findings reveal that SCAMP5 negatively regulates VDAC1 protein expression by regulating VDAC1 protein stability. To understand the role of VDAC1 in β‐cell apoptosis, we determined cleaved caspase‐3 levels in the vector and VDAC1‐overexpressing INS‐1 832/13 cells. As shown in Figure 6J, overexpression of VDAC1 resulted in increased cleaved caspase‐3 levels, confirming the crucial role of VDAC1 in cell apoptosis.^[^ ^11^ ^]^ Inversely, either inhibition of VDAC1 by its inhibitor DIDS (Figure 6K) or silencing of VDAC1 by siRNA (Figure S5D, Supporting Information) reduced the increased levels of cleaved caspase‐3 induced by shSCAMP5. To confirm the phenotype observed in vitro, we administered intraperitoneal injections of DIDS or saline to *Scamp5* cKO mice for two weeks and found that inhibition of VDAC1 by DIDS suppressed the activation of caspase‐3 (Figure 6L), thereby increasing the insulin‐positive cell area (Figure 6M,N) and improving glucose tolerance (Figure 6O,P). These data suggest that VDAC1 mediates the effects of SCAMP5 on β‐cell apoptosis.

**Figure 7:**
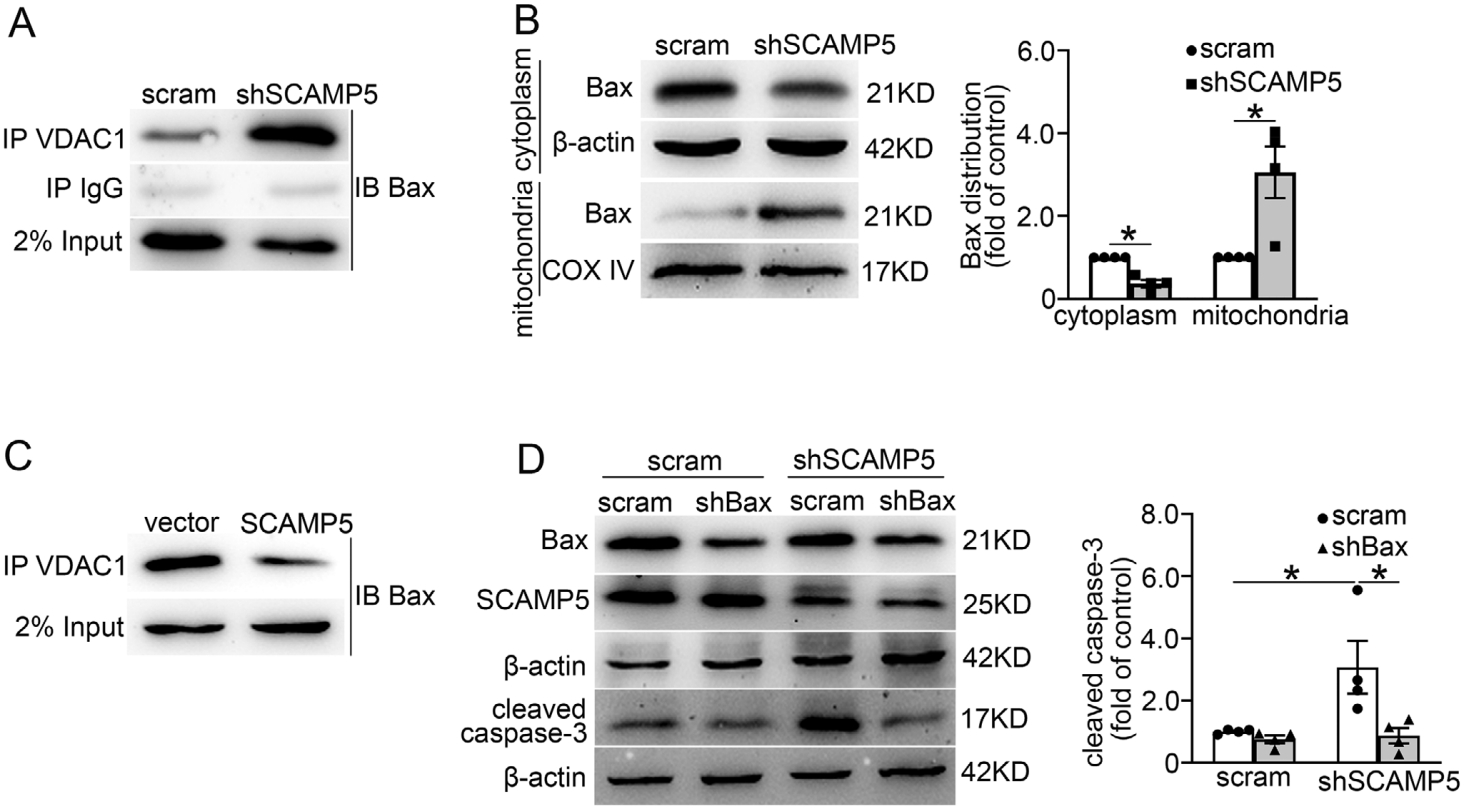
SCAMP5 regulates the interaction between VDAC1 and Bax. A) The interaction between VDAC1 and Bax in scramble and shSCAMP5 INS‐1 832/13 cells was determined by immunoprecipitation using an anti‐VDAC1 antibody, followed by a Western blot analysis with the anti‐Bax antibody. B) The subcellular localization of Bax in scramble and shSCAMP5 INS‐1 832/13 cells was determined by Western blot. β‐actin and COX IV were used as internal controls of cytosolic and mitochondrial protein, respectively. Data are means ± S.E.M., *n* = 4. *P < 0.05. C) The vector and SCAMP5‐overexpressing INS‐1 832/13 cells were cultured in a medium containing 33.3 mm glucose and 0.4 mm palmitic acid for 14 h. Then the interaction between Bax and VDAC1 in these cells was analyzed by IP using an anti‐VDAC1 antibody, followed by a Western blot with an anti‐Bax antibody. D) The protein levels of Bax, SCAMP5, and cleaved caspase‐3 in scramble and shSCAMP5 INS‐1 832/13 cells simultaneously transduced with scramble or shRNA targeted against Bax mRNA. Data are means ± S.E.M. from four independent experiments. *P < 0.05. B,D) Mann‐Whitney *U* tests were used in studies within groups.

It is reported that Bax is implicated in VDAC1‐mediated cytochrome c release from mitochondria.^[^ ^11^ ^]^ We hypothesized that Bax may be involved in VDAC1‐mediated cell apoptosis in SCAMP5‐deficient β‐cells and thus determined the interaction between VDAC1 and Bax in shSCAMP5 INS‐1 832/13 cells. This revealed that shSCAMP5 enhanced the association between VDAC1 and Bax (**Figure** **7**A). Accordingly, shSCAMP5 promoted Bax translocation from the cytoplasm to the mitochondria (Figure 7B). On the contrary, overexpression of SCAMP5 reduced the interaction between VDAC1 and Bax (Figure 7C). To understand the role of Bax in shSCAMP5‐induced β‐cell apoptosis, we constructed INS‐1 832/13 cells simultaneously knocked down both Bax and SCAMP5 and scrambled control cells. Western blot analysis confirmed the reduced protein levels of Bax and SCAMP5 (Figure 7D). Noticeably, the knockdown of Bax diminished the increased cleaved caspase‐3 levels caused by shSCAMP5 (Figure 7D), emphasizing the crucial role of Bax in shSCAMP5‐induced β‐cell apoptosis.

### 2.7 ChREBP Inhibits SCAMP5 Expression in β‐Cells

**Figure 8:**
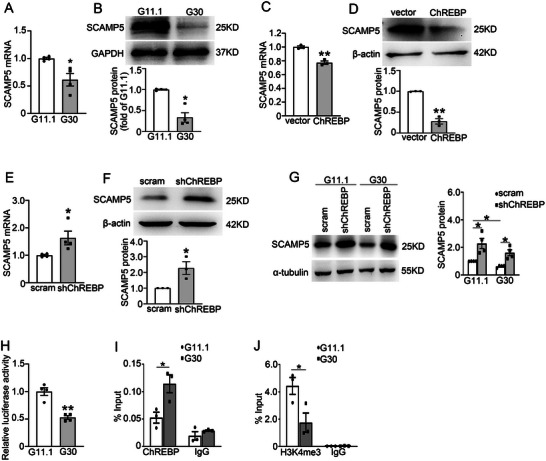
ChREBP regulates SCAMP5 expression in β‐cells. A) The mRNA and B) protein expression of SCAMP5 in INS‐1 832/13 cells that were cultured in 11.1 mm glucose (G11.1) or 30 mm glucose (G30) medium for 2 d. Data are means ± S.E.M. of four independent experiments. *P < 0.05. C) The mRNA and D) protein levels of SCAMP5 in vector and ChREBP‐overexpressing INS‐1 832/13 cells. Data are means ± S.E.M., *n* = 3. **P < 0.01. E) The mRNA and F) protein levels of SCAMP5 in the scramble and ChREBP knockdown INS‐1 832/13 cells. Data are means ± S.E.M., *n* = 3–4. *P < 0.05. G) The protein expression of SCAMP5 in the scramble and ChREBP knockdown INS‐1 832/13 cells that were cultured in G11.1 or G30 medium for 2 d. Data are means ± S.E.M., *n* = 4. *P < 0.05. H) 293T cells were transfected with a luciferase reporter driven by *Scamp5* promoter, then the cells were cultured in G11.1 or G30 medium for 2 d, followed by determination of luciferase activity. Data are means ± S.E.M., *n* = 4. **P < 0.01. I,J) INS‐1 832/13 cells were cultured in G11 or G30 medium for 2 d. ChIP assays were performed to analyze I) the occupancy of ChREBP and J) the levels of H3K4me3 at the *Scamp5* promoter. Data are means ± S.E.M., *n* = 3. *P < 0.05. The *P* values were calculated using an C–F,H–J) unpaired t‐test or A,B,G) Mann‐Whitney *U* test.

ChREBP is known to mediate gene expression changes induced by high glucose.^[^ ^5^ ^]^ To elucidate the mechanisms underlying the reduced SCAMP5 expression in diabetic conditions, we investigated the impact of ChREBP activation by high glucose on SCAMP5 expression. Consistent with the diminished expression of SCAMP5 in diabetic islets, prolonged high glucose exposure resulted in decreased SCAMP5 mRNA (**Figure** **8**A) and protein (Figure 8B) expression, respectively. We further probed into the role of ChREBP in regulating SCAMP5 expression by overexpressing or knocking down ChREBP in INS‐1 832/13 cells. This revealed that overexpression of ChREBP reduced both mRNA (Figure 8C) and protein (Figure 8D) expression of SCAMP5. Whereas ChREBP knockdown significantly enhanced SCAMP5 expression (Figure 8E,F). These data imply that ChREBP negatively regulates SCAMP5 expression in β‐cells. We hypothesized that ChREBP mediates the hyperglycemia‐reduced SCAMP5 expression. Supporting this opinion, the knockdown of ChREBP attenuated the inhibition of high glucose on SCAMP5 protein expression (Figure 8G). To comprehend the mechanism by which ChREBP regulates SCAMP5 expression, we constructed a luciferase reporter driven by the *Scamp5* promoter and evaluated the luciferase activity. The data demonstrated that activation of ChREBP by high glucose inhibited *Scamp5* promoter activity (Figure 8H). Moreover, ChIP assay revealed that high glucose promoted ChREBP binding to the promoter of *Scamp5* (Figure 8I) and reduced the level of H3K4me3, a marker associated with gene activation, at this promoter region (Figure 8J).

## 3 Discussion

In this study, we demonstrate that SCAMP5 expression is reduced in β‐cells under diabetogenic situations, which is involved in hyperglycemia‐activated ChREBP‐mediated reduction of H3K4me3 at the *Scamp5* promoter. For the first time, we illustrate that SCAMP5 is required for insulin secretion. Additionally, SCAMP5 inhibits diabetic β‐cell apoptosis by modulating VDAC1.

One hallmark of T2D is the loss of β‐cell mass, which is partially attributed to increased β‐cell apoptosis.^[^ ^12^ ^]^ However, the mechanisms underlying β‐cell apoptosis are not fully understood. Here, we demonstrate the essential role of SCAMP5 in diabetic β‐cell apoptosis. This view is supported by the fact that SCAMP5 expression was strikingly reduced in β‐cells under diabetic conditions. A loss of SCAMP5 triggers β‐cell apoptosis, whereas SCAMP5 overexpression suppresses diabetic β‐cell apoptosis.

Previous studies reported that SCAMPs are distributed in synaptic vesicles, the plasma membrane, and the Golgi apparatus.^[^ ^13^ ^]^ For the first time, we found that SCAMP5 is also localized in mitochondria. This localization confers SCAMP5 to regulate cytochrome c release from mitochondria to the cytoplasm. Given the essential role of cytochrome c in cell apoptosis, the release of cytochrome c regulated by SCAMP5 initiates the mechanisms of how SCAMP5 modulates β‐cell apoptosis. It is well‐established that the release of cytochrome c from mitochondria is controlled by VDAC1.^[^ ^14^ ^]^

VDAC1 is located on the outer mitochondrial membrane and is known to regulate apoptosis by forming oligomeric channels either independently or in conjunction with the pro‐apoptotic protein Bax. This oligomerization facilitates the release of cytochrome c into the cytoplasm, which in turn activates the apoptotic cascade.^[^ ^14^ ^]^ However, the precise mechanism underlying VDAC1‐mediated recruitment of Bax remains elusive. Our data illustrate that SCAMP5 functions as a novel inhibitor in this process, as evidenced by SCAMP5 deficiency enhancing Bax recruitment by VDAC1, whereas SCAMP5 overexpression prevents their interaction, thereby mitigating β‐cell apoptosis.

Additionally, the expression of VDAC1 is elevated in diabetic β‐cells.^[^ ^15^, ^16^ ^]^ This upregulation promotes β‐cell apoptosis (Figure 6J). Thus, inhibiting VDAC1 presents a potential therapeutic strategy for diabetes management.^[^ ^17^ ^]^ Our data demonstrate that SCAMP5 negatively regulates the expression of VDAC1: Deficiency of SCAMP5 upregulates, whereas elevated SCAMP5 suppresses VDAC1 expression in β‐cells. Furthermore, the inhibition of VDAC1 or the knockdown of Bax effectively blocks shSCAMP5‐induced β‐cell apoptosis. These findings underscore that both VDAC1 and Bax are key mediators in SCAMP5‐regulated β‐cell apoptosis. However, the mechanisms governing SCAMP5 and VDAC1 interaction require further studies.

We also demonstrate that SCAMP5 is required for insulin secretion. This view is supported by the fact that SCAMP5 deficiency diminishes, while increased SCAMP5 improves insulin secretion in diabetic β‐cells. SCAMP5 is shown to regulate vesicle transport and exocytosis,^[^ ^7^ ^]^ but the precise mechanisms remain unclear. Ca_V_1.2 is recognized as the primary functional subunit of the voltage‐dependent L‐type calcium channels in pancreatic β‐cells.^[^ ^10^ ^]^ Ca_V_1.2 facilitates the influx of Ca^2+^ in response to membrane depolarization, which is a critical step in the cascade of events leading to insulin exocytosis. The influx of Ca^2+^ triggers the fusion of insulin vesicles with the plasma membrane, thereby releasing insulin into the bloodstream.^[^ ^18^ ^]^ Our findings reveal that SCAMP5 positively regulates the expression of Ca_V_1.2, suggesting a mechanistic link through which SCAMP5 may influence insulin vesicle exocytosis. Additionally, given the important role of SCAMP5 in vesicle trafficking^[^ ^7^ ^]^ and cell apoptosis, it likely affects insulin secretion by regulating vesicle dynamics and β‐cell mass.

ChREBP is a glucose‐sensitive transcription factor that plays a critical role in the regulation of pancreatic β‐cell apoptosis.^[^ ^6^ ^]^ Under diabetic conditions, ChREBP is activated and translocates to the nucleus, where it mediates the expression of genes regulated by glucose. We, for the first time, demonstrated that SCAMP5 is one of the target genes regulated by ChREBP in β‐cells. This opinion is supported by the evidence that activation or overexpression of ChREBP suppressed, while ChREBP knockdown enhanced SCAMP5 expression. Notably, ChREBP knockdown diminished the inhibition of high glucose on SCAMP5 expression. Furthermore, our findings also revealed that high glucose promoted ChREBP occupancy and attenuated H3K4me3 at the *Scamp5* promoter. This would lead to decreased *Scamp5* gene expression, given that H3K4me3 facilitates gene transcription at the target DNA locus.^[^ ^19^ ^]^ However, the precise molecular mechanism through which ChREBP mediates SCAMP5 expression requires further exploration. Beyond these mechanistic uncertainties, although our data demonstrate SCAMP5 downregulation across rodent models, its role in human diabetic β‐cells warrants further validation.

In conclusion, our findings suggest that reduced SCAMP5 triggers β‐cell apoptosis and secretory dysfunction. The reduction in SCAMP5 expression may be a result of hyperglycemia‐induced ChREBP overactivation in diabetes. Hyperglycemia is likely to induce β‐cell dysfunction by regulating the ChREBP/SCAMP5 pathway. These findings not only uncover a novel way for regulating β‐cell dysfunction but also highlight SCAMP5 as a potential target for the treatment of T2D.

## 4 Experimental Section

### Animals and Isolation of Islets

Male Wistar and GK rats (aged 8 weeks) were procured from SLRC (Shanghai, China). ZDF and control rats (aged 8 weeks) were supplied by Beijing Vital River Laboratory Animal Technology (China). Male C57BLKS/JGpt (BKS) and BKS‐Leprem2Cd479/Gpt (db/db) mice (aged 5 weeks) were provided by GemPharmatech Co., Ltd. (Nanjing, China). *Scamp5^flox/flox^* mice were generated by inserting loxP sites flanking exon 4 of the *Scamp5* gene via CRISPR/Cas9 technology and were purchased from Shanghai Model Organisms Center, Inc. (Shanghai, China). To generate progeny with the genotype *Scamp5^flox/+^*‐*Ins2‐Cre*, *Scamp5^flox/flox^* mice were crossed with B6.Cg‐Tg(Ins2‐cre)25Mgn/J (Jackson Laboratory, stock no. 003573) transgenic mice. Then, the *Scamp5^flox/+^*‐*Ins2‐Cre* offspring were crossed with *Scamp5^flox/+^* mice to generate hemizygous *Scamp5^flox/flox^*‐*Ins2‐Cre* mice (*Scamp5* cKO). The littermate *Scamp5^flox/flox^* mice were used as controls. Experiments were performed with male control and *Scamp5* cKO mice (aged 8–30 weeks). These mice were fed an HFD for one month, followed by the administration of STZ (50 mg kg^−1^ body weight) via daily intraperitoneal injection for 5 d to mimic the states at advanced T2D.^[^ ^20^, ^21^ ^]^ *Pdx1*‐Cre (Avi tag‐2A‐CreERT2) mice (C57BL/6J) were obtained from Shanghai Model Organisms Center, Inc. (Shanghai, China). *Scamp5^flox/flox^* mice were bred with *Pdx1*‐Cre mice to produce offspring with the genotype *Scamp5^flox/flox^*‐*Pdx1‐Cre*. In this model, tamoxifen is employed to induce a β‐cell‐specific knockout of *Scamp5*. The experiments were conducted using 13‐week‐old male control mice and tamoxifen‐treated counterparts. The animal procedures were performed according to the Principles of Laboratory Animal Care and approved by the Shenzhen University Animal Care Committee. The pancreata from rats and mice were digested by collagenase P, and then the islets were picked up under a stereomicroscope, as described previously.^[^ ^22^ ^]^

### In Vivo Administration of AAV

The adeno‐associated virus AAV8‐SCAMP5 plasmid was generated by inserting the rat *Scamp5* coding sequence into the pAV‐Insulin1‐3xFlag vector. The AAV was packaged and provided by Vigene Biosciences Inc. (Jinan, China). To enhance islet SCAMP5 expression, GK rats were anesthetized with continuous isoflurane and injected with 50 µL of AAV8‐SCAMP5 containing ≈2×10^12^ virus particles into the common bile duct. The control rats were injected with 50 µL of the AAV vector.

### Intravenous Glucose Tolerance Test

Rats were fasted for 14 h and received glucose at a dose of 1.5 g kg^−1^ via a jugular vein catheter. Blood samples were drawn from the same catheter at 0, 5, 10, and 30 min after glucose administration. Plasma insulin levels were subsequently measured using the Insulin Ultrasensitive ELISA kit (ALPCO).

### Intraperitoneal Glucose Tolerance Test (IPGTT)

Mice or rats were fasted for 14 h prior to undergoing an intraperitoneal glucose injection, administered at a dose of 2 g kg^−1^ for mice or 1.5 g kg^−1^ for rats. Blood glucose levels were measured at 0, 15, 30, 60, and 120 min following glucose administration. To measure plasma insulin levels in mice, retro‐orbital blood samples were collected at 0, 5, 10, and 30 min following glucose administration. The plasma insulin levels were then determined using the Insulin ELISA kit (ALPCO).

### Plasmid Construction

The pCDH‐CMV‐MCS‐EF1‐Puro‐3×Flag and pCDH‐SCAMP5‐Flag plasmids were supplied by HanYi Biosciences lnc. (Guangzhou, China). The pMSCV‐VDAC1‐puro plasmid was created by subcloning the coding sequence of rat *Vdac1* into pMSCV‐puro vector with the primers presented in Table S1 (Supporting Information). The SCAMP5 luciferase reporter plasmid (SCAMP5‐Luc) was constructed by cloning the rat *Scamp5* promoter fragment ranging from −402 to +7 bp into pGL3‐basic luciferase reporter vector (Promega) using the primers listed in Table S1 (Supporting Information). The shBax plasmid was generated by inserting the shRNA (TRCN0000273037) targeted against rat Bax mRNA into pLKO.1‐puro vector.

### Cell Lines

INS‐1 832/13 cells were cultured in RPMI1640 medium as described.^[^ ^22^ ^]^ To establish a SCAMP5‐overexpressing cell line, the pCDH‐CMV‐MCS‐EF1‐Puro‐3×Flag plasmid (vector) or pCDH‐SCAMP5‐Flag plasmid (SCAMP5), along with packaging plasmids psPAX2 and pMD2.G, were co‐transfected into 293T cells to produce recombinant lentiviruses. The resulting lentiviruses were then used to infect INS‐1 832/13 cells, followed by selection with 3 µg mL^−1^ puromycin for 1 week. To overexpress VDAC1 in INS‐1 832/13 cells, recombinant retroviruses were produced by co‐transfecting 293T cells with the plasmids pMSCV‐puro (vector) or pMSCV‐VDAC1‐puro (VDAC1), along with packaging plasmids VSVG and PCPG. The generated retroviruses were used to infect INS‐1 832/13 cells, followed by selection with puromycin.

For the knockdown of ChREBP, SCAMP5, or Bax, either shChREBP (RMM3981‐201916728), shSCAMP5 (RMM3981‐201820435), shBax plasmid, or a nontargeting scramble control plasmid, in combination with the packaging plasmids psPAX2 and pMD2.G, was co‐transfected into 293T cells to generate recombinant lentiviruses. INS‐1 832/13 cells were subsequently infected with the generated lentiviruses and selected using puromycin.

### Cell Counting

INS‐1 832/13 cells were plated in 6‐well plates at a density of 1×10⁵ cells per well and cultured for 2 to 4 d, as specified. Then the cells were digested with 0.25% trypsin. A single‐cell suspension was generated and subsequently counted using a hemocytometer.

### Western Blot Analysis

The islets or INS‐1 832/13 cells were incubated with lysis buffer (50 mm Tris‐HCL (pH 7.6), 150 mm NaCl, 1% NP‐40, 10% Glycerol, 1 mm EDTA) supplemented with 1% protease inhibitor cocktail (Epizyme Biotech, China) for 10 minutes on ice, followed by centrifugation at 12000 rpm for 10 min at 4 °C. The supernatants were subjected to a Western blot assay. The following antibodies were used: SCAMP5 (Proteintech, 30328‐1‐AP), CACNA1C (Ca_V_1.2) (Proteintech, 21774‐1‐AP), cytochrome c (Cell signaling, #11940), caspase‐9 (Proteintech, 66169‐1‐Ig), COX IV (Proteintech, 66110‐1‐Ig), Bax (Proteintech, 60267‐1‐Ig), cleaved caspase‐3 (Cell Signaling, #9661), VDAC1 (Proteintech, 66345‐1‐Ig), GAPDH (Cell Signaling, #5174), α‐tubulin (Santa Cruz, sc‐8035) and β‐actin (Sigma, A5441). The densities of the immunoblot bands were determined by Gel‐Pro Analyzer 4.0 software.

### Co‐IP

The Co‐IP assay was performed following the method outlined previously.^[^ ^23^ ^]^ Briefly, 1 mg of cell lysate was incubated overnight at 4 °C with 3 µg of VDAC1 or IgG (Abcam, ab18413) antibodies. The resulting complexes were immunoprecipitated using 60 µl of protein A agarose beads for 3 h at 4 °C and subsequently washed with various wash buffers. The immunoprecipitated supernatants were then separated by SDS‐PAGE and analyzed through immunoblotting using Flag (Proteintech, 20543‐1‐AP) or Bax (Proteintech, 50599‐2‐Ig) antibodies.

### Real‐Time PCR (qPCR)

Real‐time PCR was performed as reported.^[^ ^24^ ^]^ The specific primers used in real‐time PCR are listed in Table S2 (Supporting Information). The target gene mRNA was normalized to the internal control of β‐actin.

### Insulin Secretion

Insulin secretion was performed as described.^[^ ^22^ ^]^ Briefly, 1 × 10^5^ INS‐1 832/13 cells or 10 islets per well were seeded in a 24‐well plate. The cells were preincubated in 1 mL glucose‐free KRB buffer for 1 h, followed by stimulation with 1 mL KRB buffer containing 2.8 mmol L^−1^ glucose or 16.8 mmol L^−1^ glucose in the absence or presence of 1 µm Bay K8644 for 30 min as indicated, respectively. The insulin levels were determined using the Insulin Ultrasensitive ELISA kit (ALPCO).

### Immunofluorescence

To assess the co‐localization of SCAMP5 and VDAC1, INS‐1 832/13 cells were fixed with paraformaldehyde and permeabilized using Triton X‐100. The cells were subsequently incubated overnight at 4 °C with primary antibodies against SCAMP5 and VDAC1, followed by incubation with fluorescent secondary antibodies. Images were captured using a ZEISS LSM 510 confocal laser scanning microscope. To evaluate the localization of SCAMP5 within mitochondria, INS‐1 832/13 cells were co‐stained with SCAMP5 antibody and MitoTracker, with imaging performed on the same confocal microscope. Additionally, pancreatic sections were stained for insulin and DAPI. Fluorescence imaging was performed using a ZEISS Axio Observer 3 fluorescent microscope. Quantitative analysis of insulin‐positive and DAPI‐stained regions was conducted using ImageJ software, with the β‐cell area calculated as the ratio of the insulin‐positive area to the DAPI‐stained area.

### TUNEL Assays

TUNEL assay was performed as described.^[^ ^23^ ^]^ Briefly, INS 832/13 cells were incubated with the TUNEL mixture for 30 min at 37 °C. Then the cells were stained with DAPI to visualize all cells. TUNEL‐positive cells were counted and normalized to total β‐cells.

### Mitochondrial Transmembrane Potential (△*Ψm*) Measurement

The **△** *Ψm* of INS‐1 832/13 cells was determined using the JC‐1 Mitochondrial Membrane Potential Assay Kit (Beyotime Biotechnology, China). Cells were incubated with a diluted JC‐1 dye (1:200) at 37 °C for 20 min, and cell‐associated fluorescence was analyzed using the Axio Observer 3 fluorescence microscope.

### Flow Cytometry

The scramble and shSCAMP5 INS‐1 832/13 cells were exposed to 0.25% trypsin for digestion. Subsequently, the cells were harvested and incubated for 20 minutes at room temperature in 200 µL of a solution containing Propidium Iodide (PI) and Annexin V. After the incubation period, the samples were immediately analyzed using a flow cytometer (Beckman) to evaluate cell apoptosis.

### SCAMP5 Promoter Reporter Assay

293T cells were transfected with SCAMP5‐Luc and Renilla luciferase plasmids and cultured in 11.1 mm or 30 mm glucose medium for 48 h. Subsequently, *Scamp5* promoter activity was determined by utilizing the Dual‐Luciferase Reporter Assay kit (Promega) according to the manufacturer's instructions. The Renilla luciferase was chosen for the normalization of luciferase activity.

### Chromatin Immunoprecipitation Assay (ChIP)

ChIP assays were performed as described.^[^ ^24^ ^]^ Chromatin was immunoprecipitated with antibodies (3 µg) against ChREBP (Novus, NB400‐135) or Tri‐Methyl‐Histone H3 (Lys4) (Cell Signaling, #9751), respectively. Final DNA extractions were sequenced from ‐334 to ‐170 bp in the *Scamp5* promoter using the primers listed in Table S3 (Supporting Information).

### Statistical Analysis

Data were expressed as mean ± standard error based on results from at least three animals or experimental replicates. Statistical analysis was performed using SPSS 20.0 software. For comparisons between two groups, either an unpaired two‐tailed t‐test or the Mann‐Whitney *U* test was employed, whereas evaluations involving more than two groups were carried out with one‐way ANOVA followed by the least significant difference (LSD) post‐hoc test. A *P* value of less than 0.05 was considered statistically significant.

## Conflict of Interest

The authors declare no conflict of interest.

## Author Contributions

Y.Z., D.T., C.Y., C.L., and Y.Y. contributed equally to this work. X.K., Y.Z., D.T., C.Y., C.L., Y.Y., M.H., and Y.C. performed the experiments. X.K., Z.M., Y.Y., and X.M. designed the experiments. X.K. analyzed data and prepared the manuscript. X.K. is the guarantor of this work and takes responsibility for the integrity and accuracy of the data analysis.

## Supporting information



Supporting Information

## Data Availability

The data that support the findings of this study are available from the corresponding author upon reasonable request.
