# Supplementary material for: Deficiency of SCAMP5 Triggers Pancreatic β‐Cell Secretory Dysfunction and Apoptosis
Source: Adv Sci (Weinh). 2025 Sep 15;12(45):e03072. doi: 10.1002/advs.202503072 (PMC12677586; doi:10.1002/advs.202503072)
Supplement: Supplementary file 1 — Supporting Information [file ADVS-12-e03072-s001.pdf]

SUPPLEMENTAL MATERIALS

Supplemental Materials

Table S1. List of Primers Used for PCR

Gene	Primers
Scamp5 promoter	Forward 5'-AACATTTCTCTATCGATAGGTACCCTGAGCCATTTCTCCAGCCC-3'
	Reverse 5'-CGTAAGAGCTCGGTACCGCTCCCGGATTGGGGG-3'
Vdac1	Forward 5'-GATCTCTCGAGGTTAACGATGGCTGTGCCTCCCAC-3'
	Reverse 5'-TCCCCTACCCGGTAGTTATGCTTGAAATTCCAGTCCTAAACCAAGC-3'

Table S2. List of Primers Used for Real-Time PCR

Gene	Primers
Scamp5	Forward 5'-GGCAGACATCCCTCCTCA-3'
	Reverse 5'-TGTAAATGGGCCGAAACC-3'
Cacna1c	Forward 5'-GTTAGCGTGTCCCTCATCT-3'
(Ca_V1.2)	Reverse 5'-ATTCTGCCTCCGTCTGTT-3'
Vdac1	Forward 5'-CTTGGCTATGAGGGTTGG -3'
	Reverse 5'-CCTGATACTTGGCTGCTATT-3'
Actin	Forward 5'-GTAAAGACCTCTATGCCAACA-3'
(beta)	Reverse 5'-GGACTCATCGTACTCCTGCT-3'

Table S3. List of Primers Used for ChIP Assay

Gene	Primers
Scamp5 promoter	Forward 5'-GAGATTGCTGTGGAGGTT-3'<br>Reverse 5'-CAGGGTGCTAGATGTTCG-3'

Supplementary Experimental Section

VDAC1 siRNA transfection: For downregulation of VDAC1, both scramble and shSCAMP5 INS-1 832/13 cells were transiently transfected for three days with either negative control siRNA or siRNA (5'-GGACUGGAAUUUCAAGCAUTT-3') targeted against VDAC1 mRNA using lipofectamine® RNAiMAX Reagent (Invitrogen, USA) according to the manufacturer's protocol.

Supplementary Figure Legends

Figure S1. SCAMP5 protein expression in scramble and shSCAMP5 INS-1 832/13 cells.

The SCAMP5 protein levels were examined in the scramble and shSCAMP5 INS-1 832/13 cells. β-actin was used as a loading control. Data are mean ± S.E.M., *n* = 6. $^{**}P<0.01$. The *P* value was determined using the Mann-Whitney *U* test.

Figure S2. Assessments of IPGTT, blood glucose levels, and body weight in both control and *Scamp5* knockout mice.

(**A**) SCAMP5 protein expression was determined in islets isolated from control and *Scamp5* cKO mice.

(**B**) IPGTT was conducted on $Scamp5^{flox/+}$-*Ins2-Cre* and $Scamp5^{flox/flox}$-*Ins2-Cre* mice. Data are mean ± S.E.M., *n* = 6-7 mice per group. $^{*}P< 0.05$.

(**C**) $Scamp5^{flox/flox}$-*Pdx1-Cre* mice were intraperitoneally injected with either a corn oil vehicle or tamoxifen at a dose of 100 mg/kg for 5 consecutive days. Subsequently, an IPGTT was performed in these mice. Data are mean ± S.E.M. from six mice per group. $^{*}P< 0.05$, $^{**}P< 0.01$.

(**D, E**) Measurements of non-fasting blood glucose (**D**) and body weight (**E**) were examined in control and *Scamp5* cKO mice at 20 weeks of age. Data are means ± S.E.M., *n* = 4-9 mice per group.

Statistical significance was determined by the Mann-Whitney *U* test (**B**) or unpaired t-

test (**C**).

Figure S3. SCAMP5 protein expression in GK-vehicle and GK-AAV8-SCAMP5 rats.

(**A**) SCAMP5 protein levels were examined in islets isolated from GK-vehicle and GK-AAV8-SCAMP5 rats. β-actin was used as an internal control. Data are means ± S.E.M., $n = 7$ rats per group. **$P< 0.01$ by Mann-Whitney *U* test.

(**B**) SCAMP5 protein levels were determined in pancreatic islets, liver, and soleus muscle tissue from both vehicle-treated and AAV8-SCAMP5-treated GK rats. β-actin was used as an internal control.

Figure S4. Effect of SCAMP5 overexpression on STZ-induced caspase-3 activation.

(**A**) SCAMP5 protein expression was measured in INS-1 832/13 cells transduced with either vector or Flag-tagged SCAMP5. α-tubulin was used as an internal control.

(**B**) The vector and SCAMP5-overexpressing INS-1 832/13 cells were treated with 300 μmol/L STZ for 15 hours. Cleaved caspase-3 levels were determined by Western blot analysis. Data are means ± S.E.M., n =3-5. *$P< 0.05$, **$P< 0.01$. Data were analyzed by the Mann-Whitney *U* test.

Figure S5. Knockdown of SCAMP5 promotes caspase-9 activation and enhances VDAC1 protein stability in INS-1 832/13 cells.

(**A**) Cleaved caspase-9 and caspase-9 levels were determined by Western blot analysis. Data are means ± S.E.M., $n = 6$. **$P< 0.01$.

(**B**) VDAC1 mRNA levels were evaluated in scramble and shSCAMP5 INS-1 832/13 cells. Data are means ± S.E.M., *n* = 6.

(**C**) After incubation with 10 μmol/L MG-132 or DMSO for 2 hours, Western blot analysis was conducted to determine VDAC1 protein levels in scramble and shSCAMP5 INS-1 832/13 cells. Data are means ± S.E.M., *n* = 5. $^{**}P < 0.01$.

(**D**) Scramble and shSCAMP5 INS-1 832/13 cells were transiently transfected with negative control siRNA (siNC) or siRNA targeted against VDAC1 (siVDAC1) for 3 days. Then, the VDAC1 and cleaved caspase-3 protein levels were determined by Western blot analysis. Data are means ± S.E.M., *n* = 3. $^{**}P < 0.01$.

P values were calculated by the Mann-Whitney *U* test (**A**, **C**) or one-way ANOVA with LSD post-hoc test (**D**).

Fig.S1

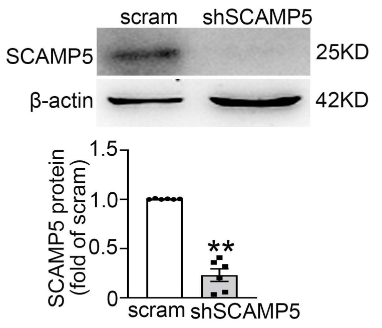
scram
shSCAMP5
SCAMP5
25KD
β-actin
42KD
SCAMP5 protein
(fold of scram)
1.5
1.0
0.5
0
**
scram
shSCAMP5

Fig.S2

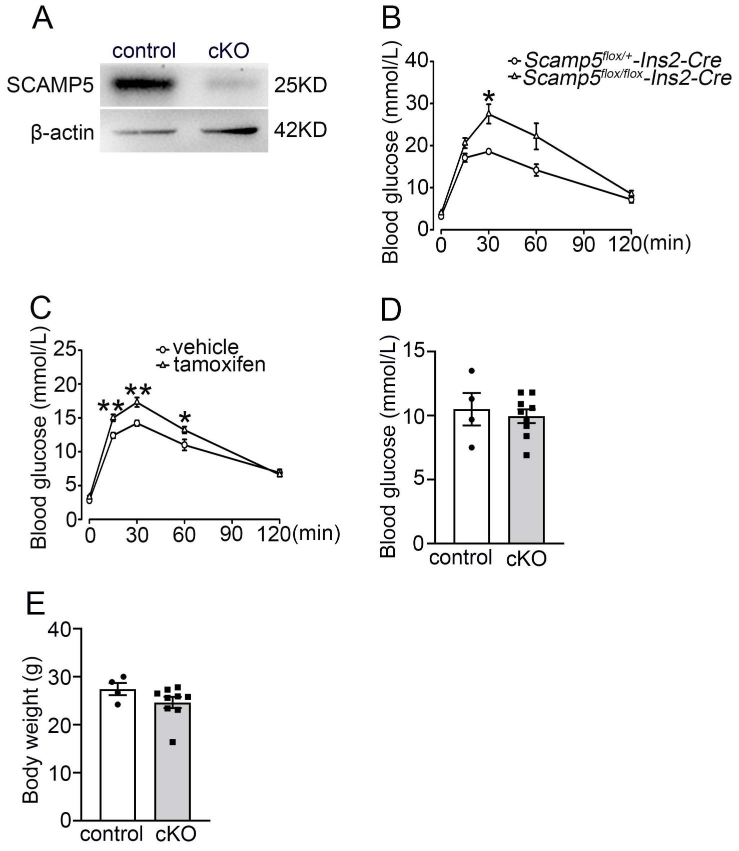
A
control
cKO
SCAMP5
25KD
β-actin
42KD
B
Scamp5flox/+-Ins2-Cre
Scamp5flox/flox-Ins2-Cre
Blood glucose (mmol/L)
(min)
C
vehicle
tamoxifen
Blood glucose (mmol/L)
(min)
D
Blood glucose (mmol/L)
control
cKO
E
Body weight (g)
control
cKO

Fig.S3

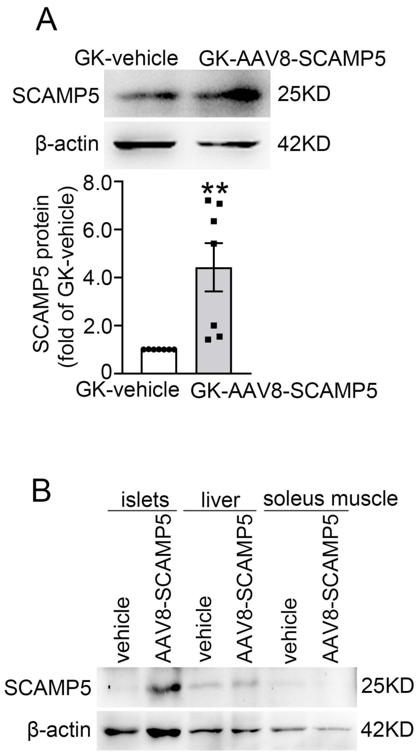
A
GK-vehicle
GK-AAV8-SCAMP5
SCAMP5
25KD
β-actin
42KD
SCAMP5 protein (fold of GK-vehicle)
8.0
6.0
4.0
2.0
0
**
GK-vehicle
GK-AAV8-SCAMP5
B
islets
liver
soleus muscle
vehicle
AAV8-SCAMP5
vehicle
AAV8-SCAMP5
vehicle
AAV8-SCAMP5
SCAMP5
25KD
β-actin
42KD

Fig.S4

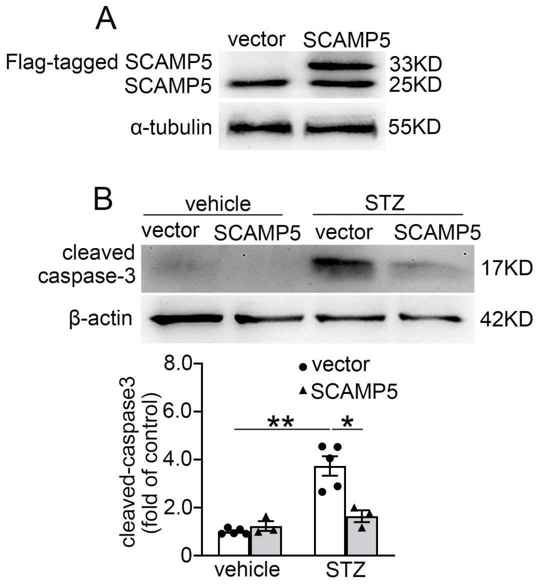

A
vector
SCAMP5
Flag-tagged SCAMP5
SCAMP5
33KD
25KD
α-tubulin
55KD
B
vehicle
STZ
vector
SCAMP5
vector
SCAMP5
cleaved caspase-3
17KD
β-actin
42KD
cleaved-caspase3 (fold of control)
8.0
6.0
4.0
2.0
0
vector
SCAMP5
**
*
vehicle
STZ

Fig.S5

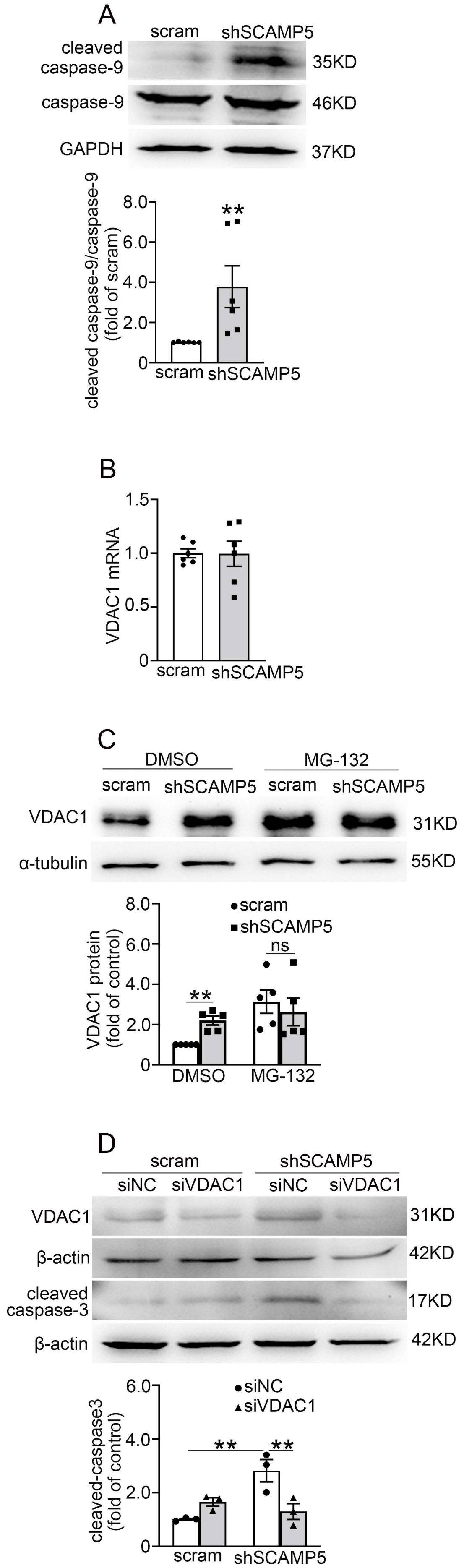
A
scram
shSCAMP5
cleaved caspase-9
35KD
caspase-9
46KD
GAPDH
37KD
cleaved caspase-9/caspase-9 (fold of scram)
8.0
6.0
4.0
2.0
0
**
scram
shSCAMP5
B
VDAC1 mRNA
1.5
1.0
0.5
0
scram
shSCAMP5
C
DMSO
MG-132
scram
shSCAMP5
scram
shSCAMP5
VDAC1
31KD
α-tubulin
55KD
VDAC1 protein (fold of control)
8.0
6.0
4.0
2.0
0
scram
shSCAMP5
ns
**
DMSO
MG-132
D
scram
shSCAMP5
siNC
siVDAC1
siNC
siVDAC1
VDAC1
31KD
β-actin
42KD
cleaved caspase-3
17KD
β-actin
42KD
cleaved-caspase3 (fold of control)
6.0
4.0
2.0
0
siNC
siVDAC1
**
**
scram
shSCAMP5
